# Development and validation of a questionnaire for analyzing real-life falls in long-term care captured on video

**DOI:** 10.1186/1471-2318-13-40

**Published:** 2013-05-01

**Authors:** Yijian Yang, Rebecca Schonnop, Fabio Feldman, Stephen N Robinovitch

**Affiliations:** 1 Technology for Injury Prevention in Seniors (TIPS) Program, Injury Prevention and Mobility Laboratory, Department of Biomedical Physiology and Kinesiology, Burnaby BC V5A 1S6, Canada; 2 School of Engineering Science, Simon Fraser University, Burnaby, BC V5A 1S6, Canada; 3 Fraser Health Authority, Surrey, BC V3R 7K1, Canada; 4 Injury Prevention and Mobility Laboratory, Department of Biomedical Physiology and Kinesiology, Simon Fraser University, 8888 University Drive, Burnaby, BC V5A 1S6, Canada

**Keywords:** Falls, Fall mechanisms, Older adults, Injuries, Long-term care, Questionnaire, Video analysis, Reliability

## Abstract

**Background:**

Falls are the number one cause of injuries in older adults, and are particularly common in long-term care (LTC). Lack of objective evidence on the mechanisms of falls in this setting is a major barrier to prevention. Video capture of real-life falls can help to address this barrier, if valid tools are available for data analysis. To address this need, we developed a 24-item fall video analysis questionnaire (FVAQ) to probe key biomechanical, behavioural, situational, and environmental aspects of the initiation, descent, and impact stages of falls. We then tested the reliability of this tool using video footage of falls collected in LTC.

**Methods:**

Over three years, we video-captured 221 falls experienced by 130 individuals in common areas (e.g., dining rooms, hallways, and lounges) of two LTC facilities. The FVAQ was developed through literature review and an iterative process to ensure our responses captured the most common behaviours observed in preliminary review of fall videos. Inter-rater reliability was assessed by comparing responses from two teams, each having three members, who reviewed 15 randomly-selected videos. Intra-rater reliability was measured by comparing responses from one team at baseline and 12 months later.

**Results:**

In 17 of the 24 questions, the percentage of inter- and intra-rater agreement was over 80% and the Cohen's Kappa was greater than 0.60, reflecting good reliability. These included questions on the cause of imbalance, activity at the time of the fall, fall direction, stepping responses, and impact to specific body sites. Poorer agreement was observed for footwear, contribution of clutter, reach-to-grasp responses, and perceived site of injury risk.

**Conclusions:**

Our results provide strong evidence of the reliability of the FVAQ for classifying biomechanical, behavioural, situational, and environmental aspects of falls captured on video in common areas in LTC. Application of this tool should reveal new and important strategies for the prevention and treatment of falls and fall-related injuries in this setting.

## Background

Falls are the cause of over 90% of hip and wrist fractures [1] and 65% of head injuries in older adults [2]. Developing improved strategies to prevent these events is an essential health priority. This is especially true for the long-term care (LTC) environment, where the complex medical status of residents causes rates of falls to be 2–3 fold higher than among community dwelling seniors [3,4], and creates unique challenges to prevention [5].

An important barrier to fall prevention is lack of objective evidence on the mechanisms of these events - how and why they occur. Our current understanding of the circumstances of falls is based on interviews or incident reports, exploring a limited set of outcomes in community-dwelling individuals [6-9]. However, most falls are unwitnessed, and accurately recalling the circumstances of falls is challenging even for young adults [10-12]. Furthermore, fallers may tend to rationalize falls as being due to an external, unavoidable cause to avoid the perception of vulnerability [10-13].

Video technology provides a means for capturing footage of real-life falls in high-risk environments such as LTC [14-16], and providing information on the biomechanical and situational aspects of falls in these settings. This information can complement clinical data (on disease diagnoses, medications, and functional status) in revealing the mechanisms of falls, and in designing and selecting prevention efforts at a population or individual level. However, this approach necessitates the development of reliable methods for extracting relevant outcomes. The present study addresses this need by developing and evaluating the inter-rater and intra-rater reliability of a 24-item questionnaire for analyzing fall mechanisms from video footage of falls captured in common areas of LTC facilities.

## Methods

### Video capture of falls

Between March 2007 and June 2010, we collected video footage of 221 falls experienced by 130 different residents from networks of digital video cameras installed in common areas (dining rooms, lounges, and hallways) in two LTC facilities in the Greater Vancouver area: Delta View Life Enrichment Centre, a 312-bed multi-level facility located in Delta, BC, and New Vista Society Care Home, a 236-bed facility located in Burnaby, BC. In both facilities, no stairs were located in the areas accessible to residents. The Delta View facility had a network of 216 digital cameras, while New Vista facility had 48 cameras. All cameras were networked to digital video recorders, which stored video data at a resolution of 640 × 480 pixels and a frame rate of 15 frames per second.

At both facilities, the occurrence of a fall (defined as “an unexpected event in which the resident comes to rest on the ground, floor, or lower lever” [17]) triggered care personnel to complete a structured incident report, as required by the Health Act of the Province of British Columbia. Members of our research team communicated daily with care personnel to review incident reports, identify falls occurring in common areas, and retrieve corresponding video footage. In 2010 at Delta View, 45% of falls occurred in common areas, of which 65% were captured on video. In 2010 at New Vista, 34% of falls occurred in common areas, of which we captured 28% on video. This study was approved by the Office of Research Ethics at Simon Fraser University and Fraser Health Authority. At the time of admission, each resident or proxy provided written permission to the facility to acquire video footage in common areas, for the purpose of resident safety. These data were shared as secondary data with our research team. We also obtained written consent from some participants to use their photographs and/or video images for the purpose of presentations or publications.

### Resident characteristics

Residents of New Vista had an average age of 81 years (SD = 13), and 67% were female. Residents at Delta View had an average age of 82 years (SD = 10), and 61% were female. Among the 15 participants included in this study, the mean age was 82 years (SD = 12), and 47% (n = 7) were women. As described previously [16], among residents captured falling who provided us with consent to access their health records, 34% had Alzheimer's disease, 13% had diabetes, 31% had hypertension, 19% had stroke, and 6% had Parkinson's disease. These prevalence data were similar to those observed among fallers not captured on video, and to the overall profile of residents at the two LTC facilities.

### Video analysis questionnaire

**Figure 1:**
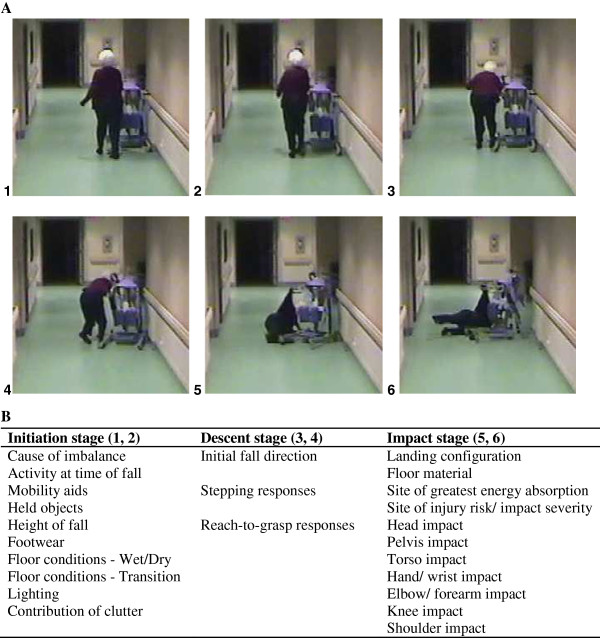
**Sample video snapshots and classification of fall characteristics: (A) sequence of images from a video recording of a real-life fall.** (**B**) Characteristics of the initiation, descent and impact stages of falls probed by the 24-item fall video analysis questionnaire (FVAQ). Note the individual shown has provided the team with written consent to include her image in publications related to this study.

Our fall video analysis questionnaire (FVAQ) included 24 primary questions probing biomechanical, situational, behavioural, and environmental aspects of falls observed in the video footage (Figure 1, Additional file 1). While falls result from interactions between physiological (intrinsic), environmental, and situational factors, video analysis itself cannot reveal physiological causes of falls (or the intentions of the faller). Instead, the FVAQ provides meaningful categorization of biomechanical features that may be important to consider, along with clinical data, in improving our understanding of the cause and prevention of falls. For each question, definitions and examples for each category (level of responses) were provided in a comprehensive instruction manual Additional file 2. We designed the FVAQ to be completed by a team of evaluators, to reduce the biases inherent in individual evaluators and allow interdisciplinary perspectives [18]. While there is no standardized approach currently for describing the mechanisms of falls, the FVAQ was based on two established conceptual models. The first model was proposed by Hayes et al. [19] and Noury et al. [20], and discusses falls as having four sequential stages: initiation, descent, impact, and post fall. The second model was proposed by Cummings et al. [21], and hypothesizes that injury risk during falls is governed by fall direction and energy-absorbing mechanisms (protective responses such as upper limp fall arrest). In selecting the responses for each question in the FVAQ, we also considered previous studies on self-reported fall circumstances (Table 1), and observations on fall characteristics emerging from our preliminary viewing and discussion of the fall videos. We also considered the approach used by Holliday and co-workers in analyzing video recordings of real-life falls captured in a Toronto area LTC facility [15]. In that study, a team reviewed each fall video to identify the activities associated with falls, environmental and behavioural contributors, balance recovery responses, impact sites, and assistive devices. Below, we summarize the FVAQ questions related to fall initiation, descent, and impact. We did not consider post-fall behaviour, such as the ability to rise after falling [20], since preliminary viewing of videos indicated that, for the vast majority of falls in common areas in LTC, residents are assisted by care staff to rise after falling. As such, a study of post-fall behaviour in this setting enters the domain of patient-care provider interactions, beyond the scope of our current study. Nor did we consider the consequences of falls.

**Table 1:** Summary of the causes and activities associated with falls in older adults

**Author (Setting)**		**Category**	
	**Cause (% of falls)**	**Activity (% of falls)**	**Other (% of falls)**
Overstall et al., [6] (Hospital and community)	Tripping (47)	After rising (6)	Miscellaneous (12)
	Drop attack (12)	Turning head (5)	
	Giddiness (9)		
	Loss of balance (8)		
Brocklehurst et al., [27] (Hospital and community)	Trip (20)	Walking	
	Lost balance (32)	Standing	
	Drop attack (23)	Postural change	
	Loss of consciousness or “other” (20)		
Lach et al., [25] (Community)	Extrinsic falls (55)		Contributing factors (% of fallers)
	• slip (27)		• sensory (28)
	• trip (21)		• shoes (26)
	• displaced center of gravity (7)		• hurrying (14)
	Intrinsic falls (31)		• external load (12)
	• mobility system failure (4)		• not common activity (11)
	• impaired balance (9)		• assistive devices (5)
	• sensory impairment (1)		• medication/alcohol (2)
	• cognitive impairment (12)		
	• impaired consciousness (6)		
	Non-bipedal stance (5)		
	• self-generated (5)		
	• support failure (<1)		
	Non-classifiable falls (9)		
Topper et al., [8] (Assisted living)	Base-of-support (BOS) perturbation (46)		Don’t know (15)
	• transfer with BOS problem (8)		
	• trip or tangle (23)		
	• slip (10)		
	Center-of-mass (COM) perturbation (28)		
	• pushed (5)		
	• collision (0)		
	• reaching, bending, turning (18)		
	• transfer without BOS problem (4)		
	No obvious perturbation (NOP) (14)		
	• loss of consciousness (3)		
	• no loss of consciousness (8)		
Cumming & Klineberg, [24] (Hospital and community)	Trip (40)	Walking (42)	Location of the fall
	Slip (10)	Being over (5)	• own residence (74)
	Leg gave way (10)	Getting up (14)	• outside (16)
	Postural change (12)	Sitting down (4)	• inside shop or club (6)
	Dizziness loss of consciousness (10)	Turing around (8)	
	Other (18)	Using stairs (3)	
Berg et al., [22] (Community)	Trip (34)	Walking on level ground (24)	Location of the fall
	Slip (25)	Walking on uneven ground (24)	• home (58)
	Misplaced step (12)	Hurrying to get work done (12)	• away from home (42)
	Loss of balance (9)	Stair ascent and decent (14)	Time of the fall
	Legs giving way (4)	Working in the yard (9)	• morning (30)
	Knocked over (4)	Carrying something heavy (9)	• afternoon (52)
	Loss of support (3)	Looking of turning (7)	• evening (14)
	Other (9)	Exercising (7); Other (7)	• night (4)
Wild et al., [26] (Hospital and community)	Fell suddenly without warning (48)	Walking (53)	Environmental hazards
	Trip, slip, miss (21)	Change of position (23)	• poor lighting (22)
	Body gave way (11)	Stair ascend or descend (13)	• stairs (13)
	Dizziness and giddiness (9)	Standing or dressing (10)	• carpets or rugs (4)
	Light-headed (6)	Fell out of chair (5)	• wet floor (4)
	Black-out (5)	Other (3)	

For fall initiation, we considered the biomechanical cause of imbalance, the activity at the time of the fall, and situational and environmental factors that have been associated with falls. These include clutter or tripping hazards, poor lighting, floor transitions, poor footwear, use of assistive devices, and held objects. Collectively, these items provide insight on “why” and “how” the fall occurred. We classified the biomechanical cause of imbalance based on the most common self-reported causes of falls in community-dwelling older adults (“trip/stumble,” “slip,” “incorrect transfer/shift of body weight,” “collapse/loss of consciousness,” and “loss of support with external object”) [6,22-26]. The FVAQ included these five categories, along with “hit/bump” (Table 2). We defined incorrect transfer/shift of body weight as loss of balance due to self-induced displacement of the body’s centre-of-gravity beyond the base of support (an “internal” rather than “external” perturbation). We classified activity at the time of falling into general categories, without consideration of the intent of the action (e.g., “walking”, as opposed to “walking to the dining room”). The most common reported activities leading to falls are walking, and transferring to or from a seated or lying position [9,22,24,26,27]. The FVAQ included these along with “standing” (Table 2).

**Table 2:** Number of response reported by the team in selecting answers for the key questions for the inter-rater and intra-rater testing (n = 15 videos)

	**Inter-rater**	**Intra-rater**	
**Question**	**Team 1 pre**	**Team 2 pre**	**Team 2 post**
	**Number of response being selected**		
Cause of imbalance			
i. Slip	0	0	0
ii. Trip/stumble	1	2	3
iii. Hit/bump	1	1	1
iv. Leg collapsed/loss of consciousness	0	0	0
v. Incorrect transfer/shift of body weight	7	5	5
vi. Loss of support with external object	6	7	6
Activity at time of fall			
i. Transferring to sitting or lying	4	4	5
ii. Transferring from sitting or lying	2	2	1
iii. Seated/wheeling in wheelchair	0	0	0
iv. Walking	4	5	5
v. Standing	5	4	4
Initial fall direction			
i. Forward	1	1	1
ii. Backward	7	7	5
iii. Sideways	6	4	4
iv. Straight down	1	3	5
Stepping response			
i. Yes	6	7	6
ii. No	9	8	9
Landing configuration			
i. Forward	1	1	1
ii. Backward	10	11	11
iii. Sideways	4	3	3
Head impact			
i. Yes	6	7	6
ii. No	9	8	9
Hand impact			
i. Yes	11	10	12
ii. No	4	5	3
Pelvis			
i. Yes	15	15	15
ii. No	0	0	0
Site of greatest injury risk			
i. Head	3	3	2
ii. Pelvis/torso/buttocks	9	9	11
iii. Upper limb	3	3	2
iv. Lower limb	0	0	0

For fall descent, we considered the initial direction of the fall and attempts to recover balance or prepare for landing (Figure 1 and Table 2). Fall direction is an important determinant of injury risk, with sideways falls causing increased risk for hip fracture [28], and forward falls causing increased risk for wrist fracture [9]. As discussed below, we considered initial fall direction separately from body configuration at landing, to account for body rotation during descent. We also investigated the appearance of balance recovery responses including stepping and grasping [29,30], which are important markers of neurological function, which, even when unsuccessful in preventing a fall, may absorb energy and reduce injury risk [31]. Finally, we examined whether active attempts were made to move the hand(s) or arm(s) into a position to arrest the fall.

For fall impact, we considered the landing configuration (forward, backward, or sideways) and the occurrence of contact to key body sites (head, pelvis, torso, hand/wrist, elbow/forearm, knee, and shoulder) (Table 2). Collectively, these items provide insight on attempts to configure the body into safe landing configuration and understanding on how the energy of the fall was absorbed or “managed.” Individual may actively modify the direction of a fall during descent [32]. Accordingly, in addition to examine the initial direction of the fall (as discussed above), we separately examined landing configuration. Impact to the head governs risk for brain injury [33], while impact to the hip or wrist dramatically increases risk for fracture at these respective sites [9,28]. However, upper extremity impact is also often protective in arresting the downward momentum of the trunk and avoiding impact and injury to the head [31]. The forces (and tissue stresses) generated during landing also depend on the number and timing of impacts to the various body parts, and on impact velocity, mass, and stiffness [34]. While recognizing it is challenging to probe these issues through a video questionnaire, we included questions on the perceived site of greatest energy absorption and the perceived site of greatest injury risk.

### Reliability testing of video analysis

Reliability testing was conducted over the course of one year. 15 fall videos were selected randomly (using a random number generator to minimize bias) from our database. Seven (47%) videos were recorded at New Vista and 8 (53%) were from Delta View. Four (27%) of the falls occurred relatively close to the camera, 5 (33%) occurred at a far distance, and 6 (40%) occurred at a moderate distance. The time interval between fall initiation (loss of balance) to fall impact ranged from about 700 ms (for a rapid trip) to 3000 ms (for a fall related to incorrect weight shifting); and the corresponding number of video frames ranged from 10 – 45. There were no major body occlusions of body segments or missing frames.

Our sample size of 15 falls was based on published guidelines for observer agreement studies [35]. We estimated a priori that (for a given question) the average percentage of agreement between the two teams would be 85 percent (or 15 percent disagreement). In order to detect a desired 90% confidence interval of between 0 and 30 percent disagreement, we calculated a minimal required sample of 15 observations.

We first evaluated inter-rater reliability by having two teams separately analyze the selected 15 videos. Each team consisted of three members, who were research assistants or graduate students trained by co-author SNR using the previously mentioned instruction manual. Team members were blinded to answers from the other team. Furthermore, team members were prevented from examining corresponding fall incident reports completed by LTC care providers (while teams would normally have this information, this created a worse-case scenario for reliability testing). Intra-rater reliability was evaluated by having one team (consisting of the same three members) re-analyze the same 15 videos one year later, while blinded to their previous answers. Each team was led by a chair, who provided instructions and recorded the team’s answers to each question. The videos were played using Windows Movie Maker (version 5.1, 2007 Microsoft Corporation). During analysis, the team members first viewed the video at normal speed, and then through frame-by-frame review while discussing and reaching consensus on the most appropriate answer to each question. We did not include “can’t tell” responses. Rather, for each question, the team was instructed to select the best available answer, along with the estimated probability (between 1-100%) of the answer being correct. On average, each fall was examined for approximately 20 minutes.

### Statistical analysis

For each question, we report the percentage of agreement between the two teams, calculated as the number of cases with the same response divided by the total number of cases, and the corresponding Cohen’s Kappa coefficient [35]. Landis and Koch [36] recommended that a Kappa value of >0.8 reflects “outstanding agreement,” 0.6-0.79 reflects “good agreement,” and 0.4-0.59 reflects “moderate agreement.” Accordingly, we considered questions with a percentage of agreement higher than 80% and a Kappa value greater than 0.6 as exhibiting “good reliability.” We also examined the association between agreement in responses and probability reported by the teams in the answer being correct using Pearson’s Correlation.

## Results

### Inter-rater reliability

19 of the 24 questions had good inter-rater reliability, with a percentage of agreement over 80% and Cohen's Kappa greater than 0.60 (Table 3). Among all questions, the average percentage of agreement was 87% and the average Kappa was 0.69. The mean probability reported by teams in selecting the correct answer ranged from 84% - 100% for one team, and from 90% - 100% for the other team. There was significant correlation between agreement in responses and probability in the answer being correct (R^2^ = 0.37; p = 0.001).

**Table 3:** Percentage of inter-rater and intra-rater agreement, Cohen’s Kappa, and mean probability confidence in selecting the answer for each question in the fall video analysis questionnaire (n = 15 videos)

**Stage of fall**	**Question**	**Inter-Rater Reliability**			**Intra-Rater Reliability**		
		**% Agreement**	**Cohen’s Kappa (95% CI)**	**Mean probability (0-100%)**	**% Agreement**	**Cohen’s Kappa (95% CI)**	**Mean Probability (0-100%)**
Initiation	Cause of imbalance	87%	0.79 (0.53-1.00)	91	93%	0.90 (0.72-1.00)	94
	Activity at time of fall	93%	0.91 (0.74-1.00)	97	93%	0.91 (0.73-1.00)	97
	Mobility aids	93%	0.89 (0.69-1.00)	95	100%	1.00 (1.00-1.00)	97
	Held objects	73%	0.33 (0.17-0.83)	97	100%	1.00 (1.00-1.00)	98
	Height of fall	100%	1.00 (1.00-1.00)	99	87%	0.71 (0.34-1.00)	99
	Footwear	67%	0.21 (0.19-0.63)	90	67%	0.29 (0.02-0.76)	90
	Floor conditions - Wet/Dry	100%	1.00 (1.00-1.00)	97	100%	1.00 (1.00-1.00)	98
	Floor conditions – Transition	100%	1.00 (1.00-1.00)	100	100%	1.00 (1.00-1.00)	100
	Lighting	93%	0.84 (0.55-1.00)	97	100%	1.00 (1.00-1.00)	99
	Contribution of clutter	47%	0.14 (0.07-0.35)	94	60%	0.24 (0.07-0.54)	96
Descent	Initial fall direction	80%	0.70 (0.40-0.99)	97	87%	0.81 (0.57-1.00)	96
	Stepping responses	93%	0.87 (0.61-1.00)	97	93%	0.87 (0.61-1.00)	96
	Reach-to-grasp responses	80%	0.44 (0.08-0.97)	94	87%	0.44 (0.15-1.00)	96
Impact	Landing configuration	93%	0.85 (0.57-1.00)	98	100%	1.00 (1.00-1.00)	97
	Floor material	100%	1.00 (1.00-1.00)	100	100%	1.00 (1.00-1.00)	100
	Perceived site of greatest energy absorption	93%	0.84 (0.55-1.00)	95	80%	0.47 (0.02-0.93)	97
	Perceived site of greatest injury risk/impact severity	67%	0.41 (0.003-0.81)	92	73%	0.47 (0.05-0.90)	94
	Head impact	80%	0.60 (0.19-1.00)	94	93%	0.87 (0.61-1.00)	95
	Pelvis impact	100%	1.00 (1.00-1.00)	99	100%	1.00 (1.00-1.00)	99
	Torso impact	80%	0.60 (0.19-1.00)	95	73%	0.41 (0.051-0.87)	98
	Hand/wrist impact	93%	0.84 (0.55-1.00)	94	87%	0.67 (0.26-1.00)	97
	Elbow/forearm impact	93%	0.84 (0.55-1.00)	96	93%	0.82 (0.47-1.00)	98
	Knee impact	93%	0.86 (0.59-1.00)	95	93%	0.86 (0.59-1.00)	96
	Shoulder impact	87%	0.70 (0.32-1.00)	96	93%	0.86 (0.59-1.00)	96

### Intra-rater reliability

18 of 24 questions had good intra-rater reliability (Table 3). The average percentage of agreement over all questions was 89% and the average Kappa was 0.74. A total of 17 of 24 questions demonstrated both good inter-rater and good intra-rater reliability. The mean probability reported by teams in selecting the correct answers ranged from 90% - 100% for the baseline analysis, and from 85% - 100% for the repeat analysis. Again, there was significant correlation between agreement in responses and probability in the answer being correct (R^2^ = 0.31; p = 0.005).

### Fall initiation

Good inter- and intra-rater reliability was observed for biomechanical cause of imbalance, activity at the time of the fall, use of mobility aids, height of the fall, and floor conditions (Table 3). However, there was poor agreement for footwear and the contribution of clutter. For held objects, the inter-rater agreement was moderate, while the intra-rater agreement was high. Incorrect weight shifting, loss of support with an external object, and tripping were the most commonly selected causes of imbalance, collectively accounting for 93% of responses (Table 2). Walking, standing, and transferring to sitting or lying were the most commonly selected activities at the time of falling, accounting for 89% of responses.

### Fall descent

Good inter- and intra-rater reliability was observed for initial fall direction and stepping responses (Table 3). There was high agreement but only moderate Kappa values for reach-to-grasp responses. The most commonly selected fall directions were backward and sideways, accounting for 42% and 31% of responses, respectively. Observable attempts to recover balance by stepping were noted in 42% of responses (Table 2).

### Fall impact

Good inter- and intra-rater reliability was observed for landing configuration and impact to the head, pelvis, hand, and knee (Table 3). Only moderate agreement was observed for torso impact, and perceived sites of greatest injury risk/impact severity. The most commonly selected landing configuration was backward (Table 2), accounting for 71% of responses. There were positive responses for impact to the head in 42% of cases, for impact to the hand(s) in 71% of cases, and for impact to the pelvis in 100% of cases. Most falls were reported to involve impacts to multiple body sites (head, torso, pelvis, knee, hand, elbow, and shoulder). In inter-rater testing, the mean number of impact sites was 4.0 (SD = 1.9) for one team, and 4.2 (SD = 1.8) for the other, with positive correlation between teams in the number of impacting sites (R^2^ = 0.84; p < 0.001).

## Discussion

Falls are the number one cause of injury in older adults, and are particularly common in LTC. Lack of objective evidence on the mechanisms of falls in this setting is a major barrier to prevention. Video capture of real-life falls can address this barrier, if valid analysis tools are available. In this study, we developed and evaluated the reliability of a comprehensive questionnaire for analyzing falls captured on video in LTC. We focused the FVAQ on the initiation, descent, and impact stages of falls [19,20] and the mechanisms that influence injury risk [21], using an iterative process to ensure our responses captured the most common behaviours observed in preliminary review of fall videos.

Our results provide strong evidence of the reliability of the FVAQ. We found that 17 of the 24 questions met our criteria for good inter-rater and intra-rater reliability. Teams rated their probability in selecting the correct answer between 84 - 100% (depending on the question), reflecting their strong confidence, and the adequacy of our video collection techniques, in identifying key features of the fall (barring significant occlusion of body parts from the camera view, which did not occur). A significant correlation existed between agreement and probability, although probability explained only 37% and 31% of the variance in inter-rater and intra-rater agreement, respectively.

In completing the FVAQ, the team often faced challenges related to camera resolution, distance between the faller and the camera. In each case, only a single camera recorded the fall. Clearly, improvements in the number and resolution of cameras should improve the reliability of most questions in the FVAQ. However, of the six poorly scoring questions, only one - type of footwear – was clearly related to video quality (e.g., distance between the faller and the camera). More complex challenges arose for other items, which might be addressed through refinements to the questions and/or instruction manual for improved clarity. For example, we observed poor reliability for contribution of clutter in causing the fall. This may more relate to the ambiguity in our definition of clutter, or the challenge of attributing casual links between falls and environmental features [25], aside from cases of obvious trips over obstacles (which made up only 13% of our sample). We observed moderate reliability for site of greatest perceived injury risk/impact severity. This may relate to difficulties in judging the injury potential of impacts to multiple body sites (on average, impact was reported to occur to 4 body sites). Reach-to-grasp responses showed good agreement but only a moderate Kappa value, perhaps due to its low frequency of occurrence creating a high probability for chance agreement [37].

The 24 questions on the FVAQ probe previously hidden aspects of falls and contribute new information to guide fall prevention efforts. For example, information on the biomechanical causes of imbalance and activities leading to falls (both of which exhibited strong reliability) helps to guide improved fall risk assessment and balance training protocols, along with efforts to reduce environmental hazards and create safer movement environments supports [16]. Information on fall severity (impacting body parts) can provide insight on injury mechanisms and help guide the design of protective padding (e.g., hip protectors [38]) and compliant “safety” flooring [39]. Attempts to prevent or lessen the injury potential of the fall (through balance recover by stepping, or arresting the fall with the upper limbs) are important neurological markers, which may also help in guiding exercise-based fall injury prevention programs.

However, there are important limitations to our study. We focused on assessing the internal reliability (reproducibility of results) of the FVAQ. Additional studies are required to examine external validity, for example by relating FVAQ responses to data from fall incident reports, observed injuries, risk for future falls, and the nature of future falls. Furthermore, we designed the FVAQ to focus on the situational and environmental context of falls in common areas of two LTC facilities (e.g., hallways, dining rooms, and living rooms). Accordingly, it may not capture the range of mechanisms of falls in bedrooms, bathrooms, and stairways, or among healthier older adults living in the community. Furthermore, the FVAQ probes a limited set of features of the built environment, behavioural factors (such as secondary attention tasks or aggression), and disease-related behaviours (such as freezing in Parkinson’s patients, or asymmetries in limb movements in stroke patients). Finally, we recognize that currently, there is limited partnering between researchers and care providers in LTC for video capture of falls. We hope that our model for data collection and analysis facilitates growth in the applications of this tool to LTC and other high risk settings, such as hospitals or senior centres [15,40]. Further “analysis packages” may build on the core template provided by the FVAQ, to probe issues such as pre-fall or post-fall behaviour, additional aspects of balance recovery or fall protective responses, or questions of known or suspected relevance to specific clinical subgroups or environments. Additional iterations should be based on a consensus process between researchers and stakeholders to agree on the right questions and response categories, and establish acceptable approaches for data collection and linking to health information.

## Conclusions

In summary, this study presents and establishes the reliability of a questionnaire for analyzing the mechanisms of falls captured on video in common areas of LTC. The FVAQ opens a window on key aspects of fall initiation, descent, and impact. When combined with health data, the FVAQ should provide researchers and clinicians with an improved understanding of the mechanisms and guidance in the prevention of falls and fall-related injuries in the high-risk LTC setting.

## Abbreviations

LTC: Long-term care; FVAQ: Fall video analysis questionnaire.

## Competing interests

The authors declared that they have no competing interests.

## Authors’ contributions

YY participated in the experimental design, data collection, data analysis, preparation and review of the manuscript. RS participated in the data collection, data analysis, preparation and review of the manuscript. FF participated in the experimental design, data collection, and review of the manuscript. SR participated in the experimental design, data collection, data analysis, preparation and review of the manuscript. All authors read and approved the final manuscript.

## Pre-publication history

The pre-publication history for this paper can be accessed here:

http://www.biomedcentral.com/1471-2318/13/40/prepub

## Supplementary Material

Additional file 1 Fall Video Analysis Questionnaire (FVAQ) – short form version. Click here for file

Additional file 2 Fall video analysis instruction manual (short version). Click here for file
