# Supplementary material for: Development and validation of a questionnaire for analyzing real-life falls in long-term care captured on video
Source: BMC Geriatr. 2013 May 1;13:40. doi: 10.1186/1471-2318-13-40 (PMC3655003; doi:10.1186/1471-2318-13-40)
Supplement: Additional file 1 — Fall Video Analysis Questionnaire (FVAQ) – short form version. [file 1471-2318-13-40-S1.pdf]

© 2013 Stephen Robinovitch, Ph.D., Simon Fraser University

technology
for injury
prevention
in seniors
tips
www.sfu.ca/tips

Fall Video Analysis Questionnaire (FVAQ) – Short Form Version

Question 1. Video details.

Record the following details on the video.

(a) Video identification code: ______________
(b) Location of fall (facility): ______________
(c) Date of fall: ______________
(d) Date of analysis: ______________
(e) Team members: ______________
(f) Team leader: ______________

Question 2. Cause of fall.

Describe the primary biomechanical cause of imbalance. Select the best answer among those listed. Estimate the percent probability (1-100%) of your answer being correct at the bottom.

i. Slip
ii. Trip/stumble
iii. Hit/bump
iv. Fell asleep/legs collapsed/loss of consciousness
v. Incorrect transfer/shift of body weight
vi. Loss of support with external object

Probability: _________

Question 3. Activity at time of fall.

Describe what the person was doing when he or she lost balance and fell. Select the best answer among those listed. Estimate the percent probability (1-100%) of your answer being correct at the bottom.

i. Lost balance while transferring from standing
ii. Lost balance while transferring from a sitting or lying position
iii. Lost balance while seated/wheeling in wheelchair
iv. Lost balance while walking
v. Lost balance while standing

Probability: _________

© 2013 Stephen Robinovitch, Ph.D., Simon Fraser University

Question 4. Mobility aids.

Describe whether a mobility aid was present at the time of the fall. Select the best answer among those listed. Estimate the percent probability (1-100%) of your answer being correct at the bottom.

i. Cane in use
ii. Wheelchair in use
iii. Walker in use
iv. Crutch in use
v. Cane visible (suspected to belong to the individual) but not being used
vi. Wheelchair visible (suspected to belong to the individual) but not being used
vii. Walker visible (suspected to belong to the individual) but not being used
viii. Crutch visible (suspected to belong to the individual) but not being used
ix. None visible belonging to the individual

Probability: _________

Question 5. Initial fall direction.

Describe the initial direction of the fall. Select the best answer among those listed. Estimate the percent probability (1-100%) of your answer being correct at the bottom.

i. Primarily forward
ii. Primarily backward
iii. Primarily sideways
iv. Straight down

Probability: _________

Question 6. Landing configuration.

Describe the configuration of the body at landing from the fall. Select the best answer among those listed. Estimate the percent probability (1-100%) of your answer being correct at the bottom.

i. Primarily forward
ii. Primarily backward
iii. Primarily sideways

Probability: _________

Question 7. Floor material.

Describe the type of floor surface the individual landed on. Select the best answer among those listed. Estimate the percent probability (1-100%) of your answer being correct at the bottom.

i. Carpet
ii. Concrete
iii. Linoleum or vinyl tile
iv. Padded mat/ compliant flooring
v. Did not land on floor

Probability: _________

Question 8. Perceived site of greatest energy absorption.

Identify the body part that absorbed the majority of energy/contact force during the impact stage of the fall. Select the best answer among those listed. Estimate the percent probability (1-100%) of your answer being correct at the bottom.

i. Head
ii. Pelvis/torso/buttocks
iii. Upper limb
iv. Lower limb

Probability: _________

Question 9. Perceived injury risk/ impact severity.

Identify the body part that appeared to be the greatest risk for injury during the impact stage of the fall. Select the best answer among those listed. Estimate the percent probability (1-100%) of your answer being correct at the bottom.

i. Head
ii. Pelvis/torso/buttocks
iii. Upper limb
iv. Lower limb

Probability: _________

© 2013 Stephen Robinovitch, Ph.D., Simon Fraser University

Question 10. Head impact.

Did impact occur to the head during the fall? Select the best answer among those listed. Estimate the percent probability (1-100%) of your answer being correct at the bottom.

i. Yes
ii. No

Probability: _________

Question 11. Pelvis impact.

Did impact occur to the pelvis during the fall? Select the best answer among those listed. Estimate the percent probability (1-100%) of your answer being correct at the bottom.

i. Yes
ii. No

Probability: _________

Question 12. Torso impact.

Did impact occur to the torso during the fall? Select the best answer among those listed. Estimate the percent probability (1-100%) of your answer being correct at the bottom.

i. Yes
ii. No

Probability: _________

Question 13. Hand/ wrist impact.

Did impact occur to the hand(s)/wrist(s) during the fall? Select the best answer among those listed. Estimate the percent probability (1-100%) of your answer being correct at the bottom.

i. Yes
ii. No

Probability: _________

© 2013 Stephen Robinovitch, Ph.D., Simon Fraser University

Question 14. Elbow/ forearm impact.

Did impact occur to the elbow(s)/ forearm(s) during the fall? Select the best answer among those listed. Estimate the percent probability (1-100%) of your answer being correct at the bottom.

i. Yes
ii. No

Probability: _________

Question 15. Knee impact.

Did impact occur to the knee(s) during the fall? Select the best answer among those listed. Estimate the percent probability (1-100%) of your answer being correct at the bottom.

i. Yes
ii. No

Probability: _________

Question 16. Shoulder impact.

Did impact occur to the shoulder(s) during the fall? Select the best answer among those listed. Estimate the percent probability (1-100%) of your answer being correct at the bottom.

i. Yes
ii. No

Probability: _________

Question 17. Stepping responses.

Did the individual attempt to recover balance by taking one or more steps? Select the best answer among those listed. Estimate the percent probability (1-100%) of your answer being correct at the bottom.

i. Yes
ii. No

Probability: _________

© 2013 Stephen Robinovitch, Ph.D., Simon Fraser University

Question 18. Held objects.

Was the individual carrying or grasping an object at time of fall? Select the best answer among those listed. Estimate the percent probability (1-100%) of your answer being correct at the bottom.

i. Yes
ii. No

Probability: _________

Question 19. Reach-to-grasp responses.

Did the individual attempt to recover balance by reaching to grasp an external object? Select the best answer among those listed. Estimate the percent probability (1-100%) of your answer being correct at the bottom.

i. Yes
ii. No

Probability: _________

Question 20. Height of fall.

Describe the height of the fall. Select the best answer among those listed. Estimate the percent probability (1-100%) of your answer being correct at the bottom.

i. Standing height
ii. Lower than standing height
iii. Greater than standing height

Probability: _________

Question 21. Footwear.

Describe the footwear worn by the resident at the time of the fall. Select the best answer among those listed. Estimate the percent probability (1-100%) of your answer being correct at the bottom.

i. Shoes
ii. Socks
iii. Slippers/ sandals
iv. Bare feet

Probability: _________

© 2013 Stephen Robinovitch, Ph.D., Simon Fraser University

Question 22. Floor conditions (Wet/Dry).

Describe whether the floor was wet or dry at the site of the fall. Select the best answer among those listed. Estimate the percent probability (1-100%) of your answer being correct at the bottom.

i. Wet
ii. Dry

Probability: _________

Question 23. Floor conditions (Transition).

Describe whether there were transitions in the colour, pattern, texture, or height of the floor at the site of the fall. Select the best answer among those listed. Estimate the percent probability (1-100%) of your answer being correct at the bottom.

i. Yes, there were transitions in the colour, pattern, texture, or height of the floor at the site of the fall
ii. No, there were no apparent transitions in the colour, pattern, texture, or height of the floor at the site of the fall

Probability: _________

Question 24. Lighting.

Describe the general lighting conditions at the site of the fall. Select the best answer among those listed. Estimate the percent probability (1-100%) of your answer being correct at the bottom.

i. Well lit (bright light)
ii. Poorly lit (dark or dim lighting)

Probability: _________

Question 25. Contribution of clutter.

Describe the apparent contribution of clutter (surrounding objects, furniture, or people) in causing the fall. Select the best answer among those listed. Estimate the percent probability (1-100%) of your answer being correct at the bottom.

i. Clutter contributed to the cause of the fall
ii. Clutter had little contribution to the cause of the fall

Probability: _________
