# Supplementary material for: Development and validation of a questionnaire for analyzing real-life falls in long-term care captured on video
Source: BMC Geriatr. 2013 May 1;13:40. doi: 10.1186/1471-2318-13-40 (PMC3655003; doi:10.1186/1471-2318-13-40)
Supplement: Additional file 2 — Fall video analysis instruction manual (short version). [file 1471-2318-13-40-S2.pdf]

© 2013 Stephen Robinovitch, Ph.D., Simon Fraser University

technology for injury prevention in seniors
tips
www.sfu.ca/tips

FALL VIDEO ANALYSIS INSTRUCTION MANUAL (SHORT VERSION)

© 2013 Stephen Robinovitch, Ph.D., Simon Fraser University

TABLE OF CONTENTS

A. INTRODUCTION TO TIPS VIDEO ANALYSIS 3
B. CONFIDENTIALITY OF DATA 3
C. GENERAL GUIDELINES FOR COMPLETING THE FALL VIDEO ANALYSIS QUESTIONNAIRE 3
D. INSTRUCTIONS AND DEFINITIONS FOR EACH ITEM IN THE QUESTIONNAIRE 4-13
QUESTION 1. VIDEO DETAILS. 4
QUESTION 2. CAUSE OF FALL. 4-5
QUESTION 3. ACTIVITY AT TIME OF FALL. 5
QUESTION 4. MOBILITY AIDS. 6
QUESTION 5. INITIAL FALL DIRECTION. 6
QUESTION 6. LANDING CONFIGURATION. 6-7
QUESTION 7. FLOOR MATERIAL. 7
QUESTION 8. PERCEIVED SITE OF GREATEST ENERGY ABSORPTION. 7
QUESTION 9. PERCEIVED INJURY RISK/ IMPACT SEVERITY. 8
QUESTION 10. HEAD IMPACT. 8
QUESTION 11. PELVIS IMPACT. 8-9
QUESTION 12. TORSO IMPACT. 9
QUESTION 13. HAND/ WRIST IMPACT. 9
QUESTION 14. ELBOW/ FOREARM IMPACT. 10
QUESTION 15. KNEE IMPACT. 10
QUESTION 16. SHOULDER IMPACT. 10-11
QUESTION 17. STEPPING RESPONSES. 11
QUESTION 18.HELD OBJECTS 11
QUESTION 19. REACH-TO-GRASP RESPONSES. 11-12
QUESTION 20. HEIGHT OF FALL 12
QUESTION 21. FOOTWEAR 12
QUESTION 22. FLOOR CONDITIONS (WET/DRY) 13
QUESTION 23. FLOOR CONDITIONS (TRANSITION) 13
QUESTION 24. LIGHTING 13
QUESTION 25. CONTRIBUTION OF CLUTTER.. 13-14

© 2013 Stephen Robinovitch, Ph.D., Simon Fraser University

A. INTRODUCTION TO TIPS VIDEO ANALYSIS

This Instruction Manual guides teams in using the Fall Video Analysis Questionnaire (FVAQ) – Short Form Version for the structured review of video footage of real-life falls in long-term care (LTC).

Falls are the number one cause of injury in older adults. An important barrier to prevention is lack of objective evidence on the circumstances of these events.

The Technology for Injury Prevention in Seniors (TIPS) program at Simon Fraser University has partnered with local long-term care (LTC) facilities, to capture video footage of falls experienced by residents, using networks of digital video cameras installed in common areas (dining rooms, lounges, and corridors).

Each fall is analyzed with a structured Fall Video Analysis Questionnaire (FVAQ), completed by a team consisting of at least three trained TIPS researchers, who meet to seek consensus on each question. This Instruction Manual assists teams in completing the FVAQ by providing definitions, interpretations, and guidelines for each question.

The team is guided through this process by a team leader, who is responsible for selecting the video footage, and making available the following materials: copies of the Questionnaire itself, copies of this Instruction Manual, a high quality LCD projector, a laptop computer containing the fall videos on the local hard drive, and any corresponding fall incident reports completed by care providers. The analysis should be completed in a quiet, private room with only team members present. In analyzing each fall, the team is able to view video footage of the fall as many times as desired.

B. CONFIDENTIALITY OF DATA

It is essential that all team members realize that the video footage represents confidential data, and the privacy of all individuals shown in the video is of the utmost importance. Accordingly, the following rules will always apply, unless you have the express permission of the TIPS Principal Investigator:

- The nature of the video images, the results of the video analysis, and information from fall incident reports should NOT be discussed outside of meetings between TIPS investigators;
- There should be NO copying or sharing of video footage (or still images of this footage);
- All team members must sign the TIPS video data confidentiality agreement.

C. GENERAL GUIDELINES FOR COMPLETING THE FALL VIDEO ANALYSIS QUESTIONNAIRE

The following general guidelines should be followed in completing the Questionnaire:

- Select the single most appropriate answer to each question.
- There are no “can’t tell” answers. Instead, the team must work together to identify the single best answer to each question, and provide their estimate of the percent probability (between 1-100%) of that answer being correct. This probability may be quite low, as in the case, for example of substantial occlusion of body parts from the camera view.
- At the start of the session, play the video file through completely. Then play desired segments of the video as many times as desired to answer each question. Although the team will likely wish to focus on only the few seconds surrounding the fall, the video should contain footage for at least 60 seconds before, and 60 seconds after the fall.
- Desired video playback software includes Windows Movie Maker, or Apple Quicktime Version 7, both of which allow control of the playback speed.

D. INSTRUCTIONS AND DEFINITIONS FOR EACH ITEM IN THE QUESTIONNAIRE

Question 1. Video details.

Record the following details on the video.

(a) Video identification code: ______________
(b) Location of fall (facility): ______________
(c) Date of fall: ______________
(d) Date of analysis: ______________
(e) Team members: ______________
(f) Team leader: ______________

Question 2. Cause of fall.

Select the answer that best describes how, from a biomechanical perspective, the resident lost balance and fell. Estimate the percent probability (1-100%) of your answer being correct. Consider the following definitions in selecting your response:

- SLIP: Loss of balance due to an apparent sliding of the foot along the floor.
- TRIP/STUMBLE: Loss of balance due to the foot or shin apparently colliding with, or becoming caught, on an object. The object may be moving or stationary, and may include, for example, another person, or the individual's other foot.
- HIT/BUMP: Loss of balance due to a body part above the knee apparently colliding with an object. The object may be moving or stationary, and may include, for example, another person.
- FELL ASLEEP/ LEGS COLLAPSED/ SUDDEN LOSS OF CONSCIOUSNESS: This is a class of falls related to loss in muscle tone, control of balance, or consciousness.
    - Select "fell asleep" when it appears most likely that the individual lost balance after dozing off.
    - Select "legs collapsed" when there is no apparent loss of consciousness, but sudden loss in muscle tone and collapsing of one or more lower extremity joints, leading to a fall.
    - Select "sudden loss of consciousness" for events that involve fainting (syncope) or seizure. Fainting is usually accompanied by loss of muscle tone (and slinking of the body to the ground). Some types of seizures also lead to loss of muscle tone, while others produce muscle rigidity.
- INCORRECT TRANSFER/ SHIFT OF BODY WEIGHT: Loss of balance due to self-induced movement of the body's centre-of-gravity beyond the base of support. Different from slip, trip/stumble, and hit/bump in the sense that the cause of imbalance is an "internal" rather than an "external" perturbation.
- LOSS OF SUPPORT WITH EXTERNAL OBJECT: Imbalance due to a sudden decrease in the supporting force from an external object (e.g., handheld object, chair, wheelchair)

Describe the primary cause of the fall. Select the best answer among those listed. Estimate the percent probability (1-100%) of your answer being correct at the bottom.

i. *Slip*
ii. *Trip/stumble*

© 2013 Stephen Robinovitch, Ph.D., Simon Fraser University

iii. Hit/bump
iv. Fell asleep/legs collapsed/loss of consciousness
v. Incorrect transfer/shift of body weight
vi. Loss of support with external object

Probability: _________

Question 3. Activity at time of fall.

Select the answer that best describes the activity the resident was engaged in at the time he or she lost balance and fell. Focus on describing what the resident was doing at the onset of imbalance. For example, if the individual rises from a sitting to standing position, reaches an upright position and then immediately displays imbalance, the correct answer to question (3) is (ii) transferring from a sitting or lying position. Consider the following definitions in selecting your response:

- TRANSFERRING FROM STANDING: Loss of balance while moving from a standing to sitting or lying position. Includes movement initiation, descent, and movement termination (impact/ weight transfer) stages.
- TRANSFERRING FROM A SITTING OR LYING POSITION: Loss of balance while moving from a sitting or lying position to a standing position, or to sitting or lying on a different surface. Includes movement initiation, rising, and movement termination stages.
- SEATED: Loss of balance from a seated configuration. Includes sitting quietly, turning, sliding off chair, or sitting and reaching.
- WALKING: Includes loss of balance while walking primarily forward, sideways, or backward. Also includes loss of balance while initiating walking, walking and turning, walking while staggering in different directions, or running. Also includes loss of balance while reaching for an object while walking.
- STANDING: Includes loss of balance while standing quietly, standing and turning, and standing and reaching.
  Note that there is no option for selecting *reaching while transferring*. If the participant was reaching at the onset of imbalance, select from 3(iii-v) to state the individual's activity while reaching or immediately prior – seated, walking, or standing.

Describe what the person was doing when he or she lost balance and fell. Select the best answer among those listed. Estimate the percent probability (1-100%) of your answer being correct at the bottom.

i. Lost balance while transferring from standing
ii. Lost balance while transferring from a sitting or lying position
iii. Lost balance while seated/wheeling in wheelchair
iv. Lost balance while walking
v. Lost balance while standing

Probability: _________

Question 4. Mobility aids.

Select that answer that best describes the type of mobility aid, if any, which was present at the time of the fall. Note the different answers for the mobility aid being “in use” (i.e., in contact with the individual) versus being visible but not in use at the time of the fall.

Describe whether a mobility aid was present at the time of the fall. Select the best answer among those listed. Estimate the percent probability (1-100%) of your answer being correct at the bottom.

i. *Cane in use*
ii. *Wheelchair in use*
iii. *Walker in use*
iv. *Crutch in use*
v. *Cane visible (suspected to belong to the individual) but not being used*
vi. *Wheelchair visible (suspected to belong to the individual) but not being used*
vii. *Walker visible (suspected to belong to the individual) but not being used*
viii. *Crutch visible (suspected to belong to the individual) but not being used*
ix. *None visible belonging to the individual*

Probability: _________

Question 5. Initial fall direction.

Select the answer that best describes the <u>initial direction of the fall just after fall initiation.</u> Note that the initial fall direction may be the same or different (due to rotation of the body during descent) than the landing configuration (Question (6)).

Describe the initial direction of the fall. Select the best answer among those listed. Estimate the percent probability (1-100%) of your answer being correct at the bottom.

i. *Primarily forward*
ii. *Primarily backward*
iii. *Primarily sideways*
iv. *Straight down*

Probability: _________

Question 6. Landing configuration.

Select the answer that best describes the <u>configuration of the body at landing from the fall</u>, based on the aspect of the body that comes into primary contact with the floor (posterior = backward, anterior = forward, lateral = sideways). Note that the landing configuration may be the same or different (due to rotation of the body during descent) than the initial fall direction (Question (5)). Regard landing in a “seated” position on the buttocks as “backward,” and landing in a kneeling position as “forward.” Neglect rolling of the body after impact, when selecting the landing configuration. However, if there are several impact stages (e.g., the body impacts a chair or the wall and then the floor), the final impact (on the floor) should be considered as “landing”.

© 2013 Stephen Robinovitch, Ph.D., Simon Fraser University

Describe the configuration of the body at landing from the fall. Select the best answer among those listed. Estimate the percent probability (1-100%) of your answer being correct at the bottom.

- *i. Primarily forward*
- *ii. Primarily backward*
- *iii. Primarily sideways*

Probability: _________

Question 7. Floor material.

Select the answer that best describes the floor surface the individual landed on. If the individual landed on two or more surfaces, choose the surface that absorbed the main impact of the fall. Select option (v) if the body did not impact the floor (but only furniture, wall, other person, etc.).

Describe the type of floor surface the individual landed on. Select the best answer among those listed. Estimate the percent probability (1-100%) of your answer being correct at the bottom.

- *i. Carpet*
- *ii. Concrete*
- *iii. Linoleum or vinyl tile*
- *iv. Padded mat/ compliant flooring*
- *v. Did not land on floor*

Probability: _________

Question 8. Perceived site of greatest energy absorption.

Select the answer that best describes the body site that appeared to absorb the majority of energy or contact force during the impact stage of the fall. Note that this is different than the body site that appeared to be at greatest risk for injury from the fall (Question 9). Note also that, in this question, the "hip" is included as part of PELVIS/TORSO/BUTTOCKS.

Identify the body part that absorbed the majority of energy/contact force during the impact stage of the fall. Select the best answer among those listed. Estimate the percent probability (1-100%) of your answer being correct at the bottom.

- *i. Head*
- *ii. Pelvis/torso/buttocks*
- *iii. Upper limb*
- *iv. Lower limb*

Probability: _________

Question 9. Perceived injury risk/ impact severity.

Select the answer that best describes the body site that appeared to be at greatest risk for injury from the fall. In the event of a "tie," select the most important vulnerable location (e.g., head over wrist, hip over knee). Note that, in this question, the "hip" is included under as part of PELVIS/TORSO/ BUTTOCKS.

Identify the body part that appeared to be the greatest risk for injury during the impact stage of the fall. Select the best answer among those listed. Estimate the percent probability (1-100%) of your answer being correct at the bottom.

- i. *Head*
- ii. *Pelvis/torso/buttocks*
- iii. *Upper limb*
- iv. *Lower limb*

Probability: _________

Question 10. Head impact.

Select the answer that best describes whether the head experienced impact during the fall.

In the event the head is blocked from view during the impact stage of the fall, do not automatically assume that head impact did not occur. Collectively consider the positions and velocities of the body segments during the visible portion of the fall, and select the most probable answer.

Make sure to separate "impact of the head during the fall" from post-fall contact of the head with the ground. For example, the faller may avoid head impact during the fall, but then later roll onto his or her back, and lie with the head on the ground. However, this does not constitute head impact.

Did impact occur to the head during the fall? Select the best answer among those listed. Estimate the percent probability (1-100%) of your answer being correct at the bottom.

- i. *Yes*
- ii. *No*

Probability: _________

Question 11. Pelvis impact.

Select the answer that best describes whether the pelvis experienced impact during the fall.

In the event the pelvis is blocked from view during the impact stage of the fall, do not automatically assume that pelvis impact did not occur. Collectively consider the positions and velocities of the body segments during the visible portion of the fall, and select the most probable answer.

Make sure to separate "impact of the pelvis during the fall" from post-fall contact of the pelvis with the ground. For example, the faller may avoid pelvis impact during the fall, but then later roll onto his or her side or back, with the pelvis contacting the ground. However, this does not constitute pelvis impact.

© 2013 Stephen Robinovitch, Ph.D., Simon Fraser University

Did impact occur to the pelvis during the fall? Select the best answer among those listed. Estimate the percent probability (1-100%) of your answer being correct at the bottom.

i. *Yes*
ii. *No*

Probability: ________

Question 12. Torso impact.

Select the answer that best describes whether the torso experienced impact during the fall.

In the event the torso is blocked from view during the impact stage of the fall, do not automatically assume that torso impact did not occur. Collectively consider the positions and velocities of the body segments during the visible portion of the fall, and select the most probable answer.

Make sure to separate "impact of the torso during the fall" from post-fall contact of the torso with the ground. For example, the faller may avoid torso impact during the fall (initially landing in a sitting position or on "all fours"), but then later roll onto his or her side or back, with the torso contacting the ground. However, this does not constitute torso impact.

Did impact occur to the torso during the fall? Select the best answer among those listed. Estimate the percent probability (1-100%) of your answer being correct at the bottom.

i. *Yes*
ii. *No*

Probability: ________

Question 13. Hand/ wrist impact.

Select the answer that best describes whether the hand(s)/ wrist(s) experienced impact during the fall.

In the event the hand(s)/ wrist(s) are blocked from view during the impact stage of the fall, do not automatically assume that impact did not occur. Collectively consider the positions and velocities of the body segments during the visible portion of the fall, and select the most probable answer.

Make sure to separate "impact of the hand(s)/ wrist(s) during the fall" from post-fall contact of the hand(s)/ wrist(s) with the ground. For example, the faller may avoid hand/ wrist impact during the fall, but then later roll onto his or her back or side, with the hand(s)/ wrist(s) contacting the ground. However, this does not constitute hand/ wrist impact.

Did impact occur to the hand(s)/wrist(s) during the fall? Select the best answer among those listed. Estimate the percent probability (1-100%) of your answer being correct at the bottom.

i. *Yes*
ii. *No*

Probability: ________

Question 14. Elbow/ forearm impact.

Select the answer that best describes whether the elbow(s)/ forearm(s) experienced impact during the fall.

In the event the elbow(s)/ forearm(s) are blocked from view during the impact stage of the fall, do not automatically assume that impact did not occur. Collectively consider the positions and velocities of the body segments during the visible portion of the fall, and select the most probable answer.

Make sure to separate "impact of the elbow(s)/ forearm(s) during the fall" from post-fall contact of the elbow(s)/ forearm(s) with the ground. For example, the faller may avoid elbow/ forearm impact during the fall, but then later roll onto his or her back, with both elbows/ forearms contacting the ground. However, this does not constitute elbow/ forearm impact.

Did impact occur to the elbow(s)/ forearm(s) during the fall? Select the best answer among those listed. Estimate the percent probability (1-100%) of your answer being correct at the bottom.

i. *Yes*
ii. *No*

Probability: _________

Question 15. Knee impact.

Select the answer that best describes whether the knee(s) experienced impact during the fall.

In the event the knee(s) are blocked from view during the impact stage of the fall, do not automatically assume that impact did not occur. Collectively consider the positions and velocities of the body segments during the visible portion of the fall, and select the most probable answer.

Make sure to separate "impact of the knee(s) during the fall" from post-fall contact of the knee(s) with the ground. For example, the faller may avoid knee impact during the fall, but then later roll onto his or her front or side, with the knee(s) contacting the ground. However, this does not constitute knee impact.

Did impact occur to the knee(s) during the fall? Select the best answer among those listed. Estimate the percent probability (1-100%) of your answer being correct at the bottom.

i. *Yes*
ii. *No*

Probability: _________

Question 16. Shoulder impact.

Select the answer that best describes whether the shoulder(s) experienced impact during the fall.

In the event the shoulder(s) are blocked from view during the impact stage of the fall, do not automatically assume that impact did not occur. Collectively consider the positions and velocities of the body segments during the visible portion of the fall, and select the most probable answer.

Make sure to separate "impact of the shoulder(s) during the fall" from post-fall contact of the shoulder(s) with the ground. For example, the faller may avoid shoulder impact during the fall, but then

later roll onto his or her side, with the shoulder contacting the ground. However, this does not constitute shoulder impact.

Did impact occur to the shoulder(s) during the fall? Select the best answer among those listed. Estimate the percent probability (1-100%) of your answer being correct at the bottom.

i. *Yes*
ii. *No*

Probability: ________

Question 17. Stepping responses.

Select the answer that best describes whether, after the onset of imbalance, the individual attempted to recover balance by stepping. Make sure to only consider steps that were executed after loss of balance, and not those executed before the onset of imbalance. Therefore, the first task for the team in answering this question is to agree upon the last stable position of the individual, after which loss of balance occurred leading to the fall. If steps were taken after this instant, then the answer to this question is "yes."

Was the individual carrying or grasping an object at time of fall? Select the best answer among those listed. Estimate the percent probability (1-100%) of your answer being correct at the bottom.

i. *Yes*
ii. *No*

Probability: ________

Question 18.Held objects.

Select the answer that best describes whether the individual was grasping or carrying an object at the time the fall was initiated.

Was the individual carrying or grasping an object at time of fall? Select the best answer among those listed. Estimate the percent probability (1-100%) of your answer being correct at the bottom.

i. *Yes*
ii. *No*

Probability: ________

Question 19. Reach-to-grasp responses.

Select the answer that best describes whether the individual attempted to recover balance by reaching to grasp an external object, which was not held before the fall was initiated. (Pay close attention to distinguishing between objects held before the fall (as probed in question (19)) versus those first grasped *during* the fall.

© 2013 Stephen Robinovitch, Ph.D., Simon Fraser University

Did the individual attempt to recover balance by reaching to grasp an external object? Select the best answer among those listed. Estimate the percent probability (1-100%) of your answer being correct at the bottom.

i. *Yes*
ii. *No*

Probability: _________

Question 20. Height of fall.

Select the best answer to describe the descent distance of the fall, considering the difference in the height of the body's centre of gravity between fall initiation and landing. "Standing height" refers, for example, to falling onto a surface at foot level from a walking or standing position. "Lower than standing height" refers, for example, to falling onto a surface at foot level from a sitting, lying, or crouching position, or falling onto furniture above foot level. "Greater than standing height" refers, for example, to falling onto a surface below foot level (e.g., from a raised platform, or down stairs) from a standing or walking position.

Describe the height of the fall. Select the best answer among those listed. Estimate the percent probability (1-100%) of your answer being correct at the bottom.

i. *Standing height*
ii. *Lower than standing height*
iii. *Greater than standing height*

Probability: _________

Question 21. Footwear.

Select the answer that best describes the type of footwear being used at the time of the fall. In this question, "slippers/ sandals" refers to footwear with an open back that is easily slipped on and off the foot.

Describe the footwear worn by the resident at the time of the fall. Select the best answer among those listed. Estimate the percent probability (1-100%) of your answer being correct at the bottom.

i. *Shoes*
ii. *Socks*
iii. *Slippers/ sandals*
iv. *Bare feet*

Probability: _________

Question 22. Floor conditions (Wet/Dry).

Select the answers that best describe whether the floor conditions at the site of the fall were wet versus dry, Try to base your answers on inspection of the floor itself (do not automatically assume the floor was wet, if a nearby "wet floor" sign is visible).

Describe whether the floor was wet or dry at the site of the fall. Select the best answer among those listed. Estimate the percent probability (1-100%) of your answer being correct at the bottom.

i. *Wet*
ii. *Dry*

Probability: _________

Question 22. Floor conditions (Transition).

Select the answers that best describe whether the there were transitions in the colour, pattern, texture, or height of the floor, and displaying glare or bright reflections of light. Transitions in the colour, pattern, texture or height may consist of steps or transitions in surface material (e.g., carpet to vinyl).

Describe whether there were transitions in the colour, pattern, texture, or height of the floor at the site of the fall. Select the best answer among those listed. Estimate the percent probability (1-100%) of your answer being correct at the bottom.

i. *Yes, there were transitions in the colour, pattern, texture, or height of the floor at the site of the fall*
ii. *No, there were no apparent transitions in the colour, pattern, texture, or height of the floor at the site of the fall*

Probability: _________

Question 24. Lighting.

Select the answer that best describes the lighting conditions at the site of the fall.

Describe the lighting conditions at the site of the fall. Select the best answer among those listed. Estimate the percent probability (1-100%) of your answer being correct at the bottom.

i. *Well lit (bright light)*
ii. *Poorly lit (dark or dim lighting)*

Probability: _________

Question 25. Contribution of clutter.

Select the answer that best describes the role of clutter (surrounding objects, furniture, or people) in causing the fall. Consider, for example, whether changes to clutter may have prevented the fall.

© 2013 Stephen Robinovitch, Ph.D., Simon Fraser University

Describe the apparent contribution of clutter in causing the fall. Select the best answer among those listed. Estimate the percent probability (1-100%) of your answer being correct at the bottom.

i. *Clutter contributed to the cause of the fall*
ii. *Clutter had little contribution to the cause of the fall*

Probability: _________
